# Management of neonatal giant occipital encephalocele: Anaesthetic challenge

**DOI:** 10.4103/0019-5049.71022

**Published:** 2010

**Authors:** Vikash Goel, Neelam Dogra, Mamta Khandelwal, RS Chaudhri

**Affiliations:** Department of Anaesthesiology, S.M.S Medical College and Hospital, Jaipur, Rajasthan, India; 1 Department of Paediatric Anaesthesiology, Sir Padmapat Mother and Child Health Institute, S.M.S. Medical College, Jaipur (Rajasthan), India

**Figure 1:**
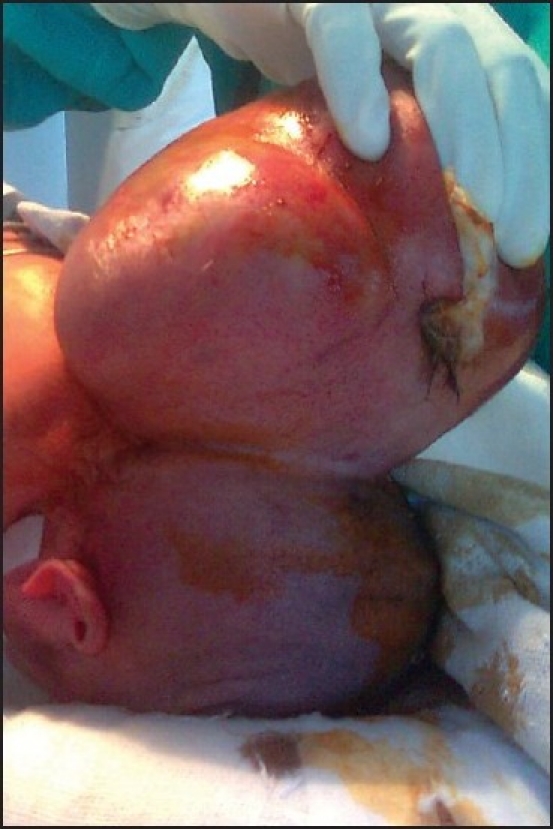
Neonate with giant occipital encephalocele

Sir,

Proper positioning is a prerequisite for successful endotracheal intubation. A giant occipital encephalocele may prevent this. We report such a case in which a two-day-old neonate weighing three kilograms presented with a large cystic swelling measuring 22 × 16 cm [Figure 1], arising from the occipital region. There was no neurological deficit and no other congenital abnormality. In the operating room, we could not place the baby supine and so anaesthesia was induced in the lateral position. Drugs used were Glycopyrrolate 3 μg/kg intravenously (i.v.), fentanyl 1.5 μg/kg i.v. and propofol 2 g/kg i.v. Muscle relaxation was achieved using succinylcholine 2 mg/kg. We attempted laryngoscopy in the lateral position, but only the tip of the epiglottis could be visualized (Cormack-Lehane Grade 3), so intubation could not be done. We continued to ventilate the baby in the lateral position with a bag and mask, using 100% oxygen. Next, we lifted the baby and placed her head beyond the edge of table with an assistant supporting it while another assistant stabilized the baby’s body, taking adequate care to support the encephalocele so as to prevent a rupture. Laryngoscopy in this position provided a better view (Cormack-Lehane Grade 2) and we intubated the trachea with a 3 mm uncuffed endotracheal tube and secured it adequately. Following this, we placed the baby carefully in the prone position. Intraoperatively, the baby was monitored vigilantly. Anaesthesia was maintained with N_2_O : O_2_ :: 50 : 50, halothane 0.6%, atracurium infusion 10 μg/kg/min and fentanyl infusion 2 μg/kg/hr. Thereafter, we proceeded uneventfully till the end of surgery.

Paediatric patients have a low functional reserve volume, and failure to intubate the trachea may result in hypoxaemia, bradycardia and even cardiac arrest. Airway pathology, improper positioning and limited neck extension can make endotracheal intubation difficult or impossible. Therefore, an alternative airway management plan should be ready prior to commencement of the procedure. This should include laryngeal mask airway of appropriate size, with high frequency jet ventilation, fiberoptic bronchoscope, a cricothyroid cannula and preparations for tracheostomy.[1] Blind nasal intubation can also be tried. Alternative approaches include placing the child supine on a platform of rolled-up blankets, awake intubation in the lateral position, or needle decompression of the encephalocele sac under sterile conditions. In patients with encephalocele, inadequate spontaneous respiration may occur due to structural derangement in the pontomedullary respiratory control center or its afferent and efferent pathways.[2,3] Aspiration may occur because of lack of pharyngeal coordination, poor sucking reflex and absent gag reflex.[2,3] If there are any concerns regarding aspiration in combination with difficult airway management, a fiberoptic awake tracheal intubation should be the technique of choice. All preparations for resuscitation should also be conducted. The patient should be turned into the prone position carefully, so that ventilation is not impeded, venous return is not compromised and all pressure points are protected. In infants with encephalocele, dysfunction of autonomic control below the level of the defect makes conservation of body temperature important.[2,4] Preparation for significant blood loss should be made because of potential bleeding from the suboccipital bone and the dural sinus. The ultimate prognosis depends on various factors, including the extent and nature of the herniated contents and associated congenital anomalies. To conclude, in case of a giant occipital encephalocele, placing the baby’s head beyond the edge of table is a promising approach to intubate the patient and may be used as an alternative to intubation in the lateral position.
